# Independent Predictive Ability of Procalcitonin of Acute Kidney Injury among Critically Ill Patients

**DOI:** 10.3390/jcm9061939

**Published:** 2020-06-21

**Authors:** Ya-Ting Huang, Min-Yu Lai, Wei-Chih Kan, Chih-Chung Shiao

**Affiliations:** 1 Department of Nursing, Camillians Saint Mary’s Hospital Luodong. No. 160, Zhongheng S. Rd., Luodong, Yilan 26546, Taiwan; frankie7451@gmail.com (Y.-T.H.); smh01603@smh.org.tw (M.-Y.L.); 2 Department of Nephrology, Department of Internal medicine, Chi-Mei Medical Center, Yongkang District, Tainan City 710, Taiwan; rockiekan@ntu.edu.tw; 3 Department of Biological Science and Technology, Chung-Hwa University of Medical Technology, Rende District, Tainan City 717, Taiwan; 4 Division of Nephrology, Department of Internal Medicine, Camillians Saint Mary’s Hospital Luodong. No. 160, Zhongheng S. Rd., Luodong, Yilan 26546, Taiwan; 5 Saint Mary’s Junior College of Medicine, Nursing and Management, No.100, Ln. 265, Sec. 2, Sanxing Rd., Sanxing Township, Yilan County 266, Taiwan

**Keywords:** acute kidney injury, infection, predictor, procalcitonin

## Abstract

It is unclear whether serum procalcitonin (PCT) levels rise in patients with acute kidney injury (AKI), and it is also unclear whether the elevation of PCT levels in this setting is independent of the existence of infection and impaired renal clearance. We conducted a retrospective study in a regional teaching hospital in Taiwan to evaluate the AKI-predictive ability of serum PCT among critically ill patients. We enrolled 330 patients (mean age, 70.5 ± 16.4 years; 57.0% men) who were admitted to the intensive care unit (ICU) from 1 July 2016, to 31 December 2016, and who had serum PCT measurement performed within 24 h after ICU admission. We used the generalized additive model and generalized linear model to evaluate the association of serum PCT levels and renal function variables. In addition, we used the multivariate logistic regression method to demonstrate serum PCT level as an independent predictor of AKI in both the non-infected patients (odds ratio (OR) = 1.38, 95% confidence interval (CI) = 1.12–1.71, *p* = 0.003) and the infected patients (OR = 1.23, 95% CI = 1.03–1.46, *p* = 0.020). In conclusion, serum PCT level at ICU admission is an independent predictor of developing AKI irrespective of infection among critically ill patients.

## 1. Introduction

Acute kidney injury (AKI) is a common but complex disorder characterized by a rapid deterioration of kidney function. AKI affects about 2 to 7% of hospitalized patients and 13 to 78% of critically ill patients [1,2,3,4], with high morbidity and mortality [5]. Over the decades, advancements in therapies for AKI have been limited, and the prognoses in AKI patients remain unsatisfactory [6]. A crucial strategy for resolving this frustrating problem is to find promising biomarkers that could early identify the onset, location, type, etiology, and severity of kidney injury [7].

Procalcitonin (PCT) is a 116-amino acid peptide that behaves as a precursor in calcium homeostasis. In the inflammatory or infectious states, serum PCT level rises rapidly within 3–4 h after onset, reaches a peak level within 6–24 h, and starts to decline after 24 h and return to a normal level by the 5th day [8,9]. As a result, PCT has been proposed as a promising marker for microbial infection and sepsis [10], as well as a useful marker to guide antibiotics therapy in critical patients with severe sepsis and septic shock [11].

PCT has ever been disclosed as a predictor of AKI in different clinical settings irrespective of the existence of infection [4,12]; nevertheless, contradicting reports appeared in the literature [3,13,14]. Besides, the existing knowledge shows that the elevated serum PCT levels in the infection or inflammatory status might be related to the impaired renal clearance of PCT in the chronic kidney disease (CKD) setting [15]. Furthermore, AKI often develops following severe infection [16,17], and is associated with a pro-inflammatory nature and a reduced waste clearance. However, it is still not clarified whether the serum PCT level elevates in AKI patients, or whether the PCT level in this situation is independent of the existence of infection and impaired renal clearance. This study aimed to prove the hypothesis that serum PCT level is an independent predictor of the development of AKI, and its predictive ability is independent of infection and impaired renal function among critically ill patients.

## 2. Materials and Methods

### 2.1. Study Design and Participants Selection

This study was reviewed and approved by the Institutional Review Board (IRB) of Saint Mary’s Hospital Luodong (# SMHIRB_105012). The study design conformed to the ethical guidelines and the Helsinki Declaration of 1975 and its revised version in 2013. The study was performed following the study protocol and relevant guidelines. The need for written informed consent was waived by the above IRB because there was neither breach of privacy nor interference with clinical practice. The data were analyzed anonymously.

The retrospective study was conducted in a regional teaching hospital in Taiwan. The inclusion criteria included adult patients who admitted to the intensive care unit (ICU) during the period from 1 July 2016, to 31 December 2016, and had serum PCT levels measured within 24 h after ICU admission. The exclusion criteria included patients less than 18 years of age, and those who had been exposed to surgeries or trauma within seven days before serum PCT measurement. We only took the data of the first hospitalization for analysis in the patients who had more than one hospitalization. After obtaining the basic and clinical data, we evaluated the association of serum PCT levels with AKI, residual renal function, and infection.

### 2.2. Measurements

The demographic data, comorbidities, clinical variables including vital signs, laboratory tests, the support of mechanical ventilators, or noninvasive positive pressure ventilators (NIPPV), the experience with cardiopulmonary resuscitation (CPR), and vasopressors support at ICU admission, as well as 30-day mortality were obtained from medical records. Several severity scores, including the acute physiology and chronic health evaluation II (APACHE II) and the sequential organ failure assessment (SOFA) score [18] at ICU admission, were calculated.

The AKI was diagnosed according to the Kidney Disease: Improving Global Outcomes (KDIGO) criteria [19]. The baseline serum creatinine (SCr) was defined as the latest SCr before the index admission. For the patients whose SCr level before admission is not available, the baseline SCr was calculated using the Modification of Diet in Renal Disease (MDRD) formula [20]. The peak SCr was defined by the highest SCr level within seven days after ICU admission. The SCr ratio denoted the ratio of peak SCr to baseline SCr, while the delta SCr presented the difference between peak SCr and baseline SCr. The estimated glomerular filtration rate (eGFR) was calculated by the MDRD formula [20]. Two physicians established the diagnosis of infection according to the laboratory tests (such as white blood cell count, urine routine, C-reactive protein), image study, culture results, and clinical presentations.

### 2.3. Quantitative Measurement of Biomarkers

All the laboratory tests were performed in the central laboratory of the hospital. The serum PCT levels were measured using the sandwich principle of an automatic electrochemiluminescent immunoassay (COBAS E411, ROCHE, Basel, Switzerland) with the analytical measurement range of 0.02–100 ng/mL and detection limit of <0.02 ng/mL.

### 2.4. Statistical Analysis

We used Scientific Package for Social Science (PASW Statistics for Windows, Version 22.0, Chicago, IL, USA: SPSS Inc.) and R 3.6.3 (R Foundation for Statistical Computing, Vienna, Austria, accessed https://www.r-project.org/) software for statistical analyses, and took a *p* ≤ 0.05 as statistically significant in all statistical analyses.

Categorical variables were expressed as case number (percentage) and compared using the chi-square test. Continuous variables experienced normality evaluation using the Kolmogorov–Smirnov test and the Shapiro–Wilk normality test [21]. The continuous variables with normal distribution were reported as mean ± standard deviation (SD) and compared using an independent *t*-test. The continuous variables with abnormal distribution were expressed as median (range) and compared using an independent *t*-test after log transformation [22] and confirmation as normal distribution by QQ plot. The Kruskal–Wallis test was used to compare the serum PCT level among patients with different AKI stages, while the Mann–Whitney U test was used to compare the serum PCT level between two groups with small sample sizes and unequal case numbers in the two groups. Furthermore, the analysis of covariance (ANCOVA) was performed using mixed linear models, with statistical control for the effects of baseline renal function and severity score, to compare the PCT levels among patients with different AKI stages and infection states.

We used the generalized additive model (GAM) and generalized linear model (GLM) to evaluate the association between the serum PCT levels and some variables (such as SCr ratio, delta_SCr, and eGFR). The trends of lines were plotted by linear regression line with a LOESS (locally estimated scatterplot smoothing) curve, which is a nonparametric technique using locally weighted regression to fit a smooth curve through points in a scatter plot.

The conditional backward stepwise model of multivariate logistic regression method was used to investigate the odds ratio (OR), 95% confidence interval (CI) and *p*-value of the independent predictors of AKI after testing collinearity statistics. Multicollinearity was defined as a variance inflation factor (VIF) value of more than 4.0 or tolerance of less than 0.2 [23]. We put relevant variables expressing demographic information, baseline renal function, and severity score along with serum PCT levels into the multivariate logistic regression to determine the independent predictors for AKI. Hosmer and Lemeshow goodness-of-fit tests were used for calibration of the model.

By using the simple logistic regression to evaluate the association of AKI and the log-transformed PCT levels, we obtained an OR of 1.412. The power subsequently calculated by G-Power of the logistic regression method in our study was 0.99 when setting α as 0.05 and OR as 1.412.

## 3. Results

**Figure 1:**
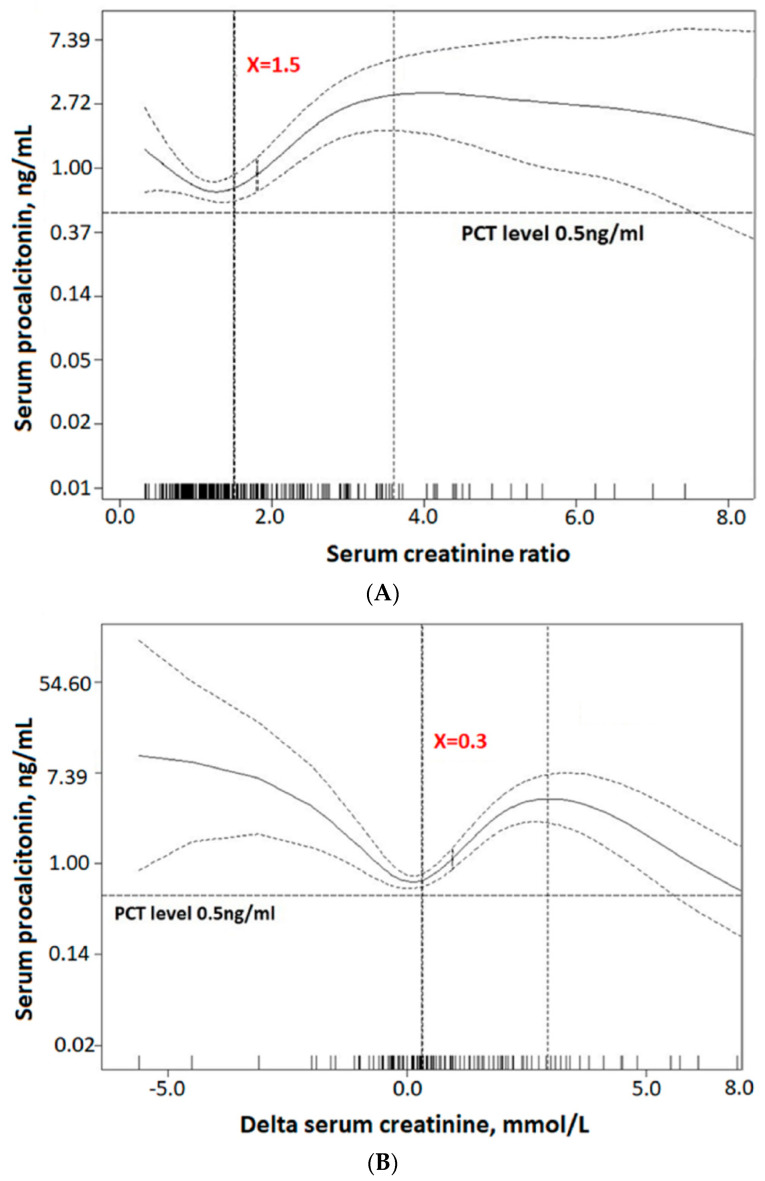
Generalized additive model plots showing the trends of PCT changes along with the increase in (**A**) SCr ratio, and (**B**) delta SCr. Abbreviations: SCr = serum creatinine; PCT = procalcitonin.

During the enrollment period, a total of 745 patients were extracted from the hospital database. After excluding 415 patients, we enrolled a total of 330 patients (mean age, 70.5 ± 16.4 years; 57.0% men). Among them, the baseline SCr values of 46 patients were estimated by the MDRD formula due to insufficient data. The GAM plots disclosed that the serum PCT levels started to increase with a SCr ratio of 1.5 (Figure 1A) and a delta SCr of 0.3 mg/dL (Figure 1B), which together indicated stage 1 AKI. Accordingly, we categorized the patients into the AKI group (*n* = 127, 38.5%) and the non-AKI group (*n* = 203, 61.5%) by the existence of AKI (stage 1 and more advanced stages) within seven days after ICU admission.

### 3.1. Basic Characteristics and Clinical Variables of the Two Groups

The basic demographic information, comorbid diseases, and most of the clinical variables at ICU admission were not significantly different between the AKI group and the non-AKI group. The only statistical differences were that the AKI group had a higher proportion of pneumonia (21.3% vs. 12.3%, *p* = 0.030) and a higher SOFA score (8.1 ± 3.6 vs. 6.2 ± 3.8 points). Besides, the AKI group had higher 30-day mortality than the non-AKI group (31.5% vs. 20.2%, *p* = 0.020). (Table 1) As for the laboratory data, the AKI group had higher serum PCT levels (2.3 (0.05, 234.6) vs. 0.5 (0.02, 242.8) ng/mL), blood urea nitrogen (BUN) (54.4 (11.0, 205.0) vs. 23.4 (5.3, 210.7) mmol/L), SCr (2.6 (0.4, 18.2) vs. 1.0 (0.3, 15.9) mmol/L), alanine aminotransferase (ALT) (36.0 (3.0, 1891.0) vs. 21.0 (1.0, 709.0) units/L, *p* = 0.002), potassium (4.4 ± 1.2 vs. 4.1 ± 1.0 mEq/L, *p* = 0.031), but lower eGFR (23.5 (1.8, 557.8) vs. 65.9 (1.3, 382.3) mL/min/1.73 m^2^) and bicarbonate (HCO3) (17.5 ± 7.4 vs. 21.2 ± 7.8 mEq/L) than the non-AKI group. (Table 2) (All *p* < 0.001 unless otherwise denoted).

### 3.2. The Association among Infection, Acute Kidney Injury and Impaired Renal Function

Regardless of the existence of infection, the serum PCT level had an increasing trend along with the worsening severity of AKI (denoted by increasing SCr ratio, Figure S1A) and the worsening residual renal function (denoted by decreasing eGFR, Figure S1B) within the clinically relevant range. The serum PCT levels were significantly higher in the infection group than in the non-infection group in the above two figures. (Both *p* < 0.001) Moreover, the PCT levels of infected patients were above 0.5 ng/mL, the cut-point indicative of systemic infection [3]. (Figure S1A,B).

**Figure 2:**
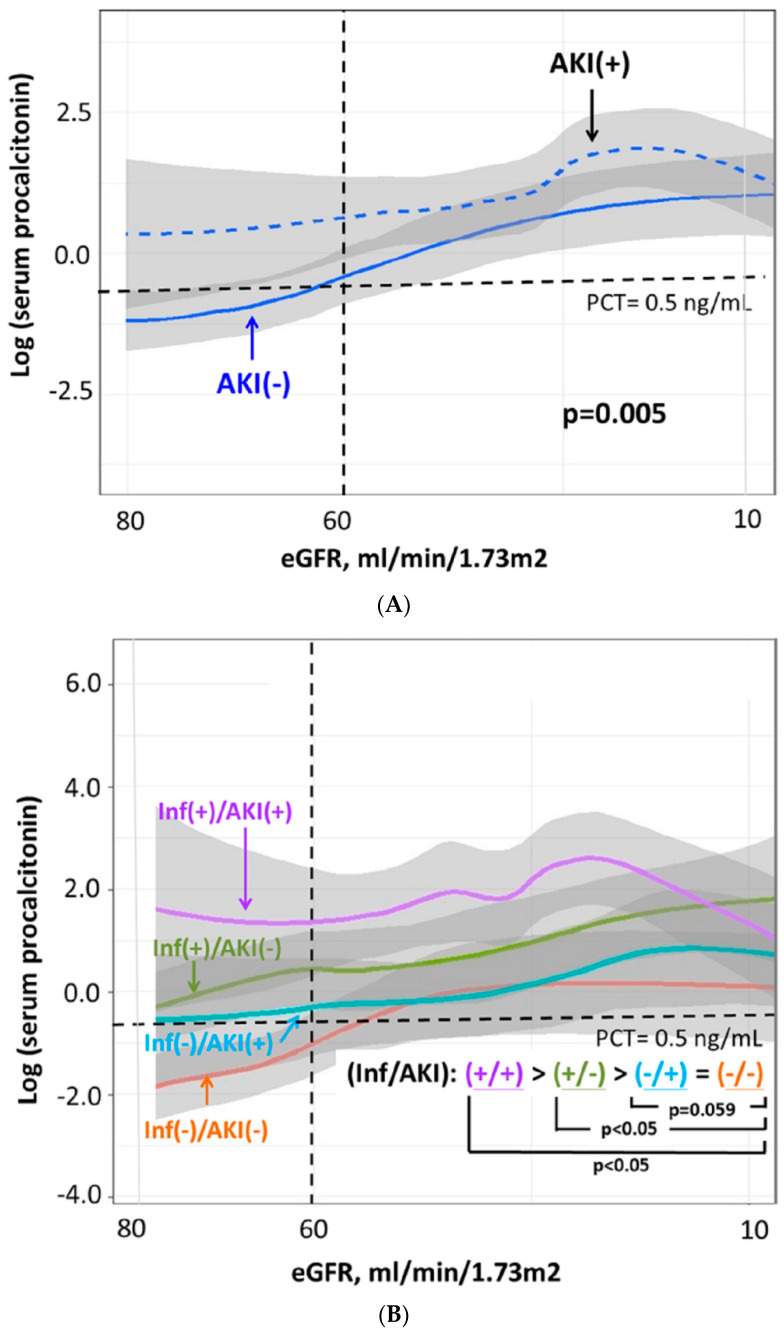
Comparisons of PCT levels among groups stratified by (**A**) AKI, and (**B**) AKI and infection. Abbreviations: AKI = acute kidney injury; eGFR = estimated glomerular filtration rate; inf = infection; PCT = procalcitonin.

Furthermore, an increasing trend of the PCT levels, along with the worsening residual renal function, could be seen in patients regardless of the existence of AKI. The serum PCT levels of the AKI group were significantly higher than those of the non-AKI group at the same corresponding eGFR levels. (*p* = 0.005) The finding could be interpreted as higher serum PCT levels in AKI patients than CKD patients (presented as non-AKI patients with eGFR < 60 mL/min/1.73 m^2^) at the same eGFR levels. (Figure 2A).

Then, we further compared the PCT levels among four groups stratified by the presence of AKI and infection. We found that the infection (+)/AKI (+) group had the highest serum PCT levels, followed along with the subsequently decreasing PCT levels in the infection (+)/AKI (−) group, the infection (−)/AKI (+) group and the infection (−)/AKI (−) group. The differences in serum PCT levels between any two groups were statistically significant, except the difference between the infection (−)/AKI (+) group and the infection (−)/AKI (−) group. (Figure 2B).

On the other hand, the median serum PCT levels were significantly higher in the AKI (+) group than in the AKI (−) group irrespective of the presence of infection. Additionally, the serum PCT levels were significantly higher in the infection (+) group than in the infection (−) group, irrespective of the presence of AKI. (All *p* < 0.001) (Figure S2).

**Figure 3:**
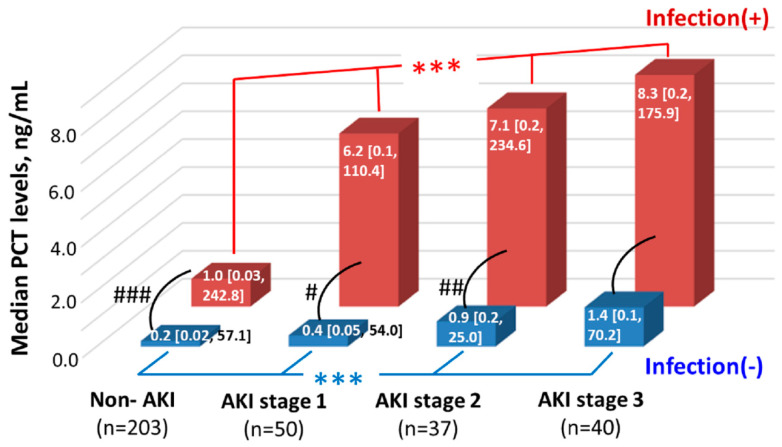
Median PCT levels among groups stratified by AKI stages and infection. Note: Data are expressed as median (range). *** denotes *p* < 0.001 in the comparisons of serum PCT levels among patients with different AKI stages using the Kruskal–Wallis test. #, ##, ### denote *p* < 0.05, <0.01, <0.001, respectively, in the comparison of serum PCT levels between infection (+) group and infection (−) group using the Mann–Whitney U test. Abbreviations: AKI = acute kidney injury; PCT = procalcitonin.

Regarding the different stages of AKI, we found that the serum PCT levels significantly and stepwise increased from the non-AKI group to the AKI stage 3 group in both infection (+) and infection (−) groups. Whereas the infection (+) group had significantly higher serum PCT levels than the infection (−) group at most of the AKI stages except stage 3. (Figure 3) Furthermore, we performed ANCOVA with log-transformed PCT as the measure of interest (dependent variable), AKI stages and infection as fixed factors, and baseline eGFR and SOFA score as covariates. The ANCOVA confirmed that with the control for baseline renal function and disease severity, serum PCT levels were statistically different among patients with different AKI stages irrespective of infection, and between patients with and without infection irrespective of the AKI stages. (Both *p* < 0.001) (Table S1).

### 3.3. The Predictive Ability of Serum Procalcitonin for Acute Kidney Injury

In the multivariate logistic regression analysis including log-transformed PCT levels along with age, gender, baseline eGFR, and SOFA score at ICU admission for adjustment, we demonstrated that the serum PCT level behaved as an independent factor for predicting AKI in the whole cohort (OR = 1.27, 95% CI = 1.12–1.43), including the non-infection patients (OR = 1.38, 95% CI = 1.12–1.71, *p* = 0.003) and the infection group (OR = 1.23, 95% CI = 1.03–1.46, *p* = 0.020). (Table 3) Hosmer and Lemeshow goodness-of-fit tests confirmed the calibration of the models of the whole cohort (chi-square = 8.44, *p* = 0.394), the non-infection patients (chi-square = 6.52, *p* = 0.588) and the infection patients (chi-square = 5.28, *p* = 0.734). (All *p* < 0.001 unless otherwise denoted).

## 4. Discussion

To the best of our knowledge, the current study is among the first few studies demonstrating the complex association of serum PT level with AKI, infection and impaired residual renal function. The strength of the current study is the comparisons of the serum PCT levels between different settings at the same residual renal function. The study had the following findings: (1) The existence of infection, the worsening residual renal function and the increasing severity of AKI respectively associated with increased serum PCT levels. (Figure 2 and Figure 3, Supplementary Figures S1 and S2) (2) The influence on serum PT levels of AKI was more significant than CKD with the same residual renal function. (Figure 2A) (3) The serum PCT level was an independent predictor for the development of AKI regardless of the existence of an infection.

### 4.1. Influence on Procalcitonin: Infection, Residual Renal Function and Acute Kidney Injury

The PCT is produced and converted to calcitonin within the thyroid C cells before releasing into the circulation, resulting in a very low (0.05 ng/mL) serum PCT levels in healthy subjects [24]. In the inflammatory state, the calcitonin production process is independent of the above regulations [24], causing a variously-degreed increase in calcitonin content in various organs [25]. Thus PCT has been recognized as a useful marker for detecting infection/inflammatory, guiding antibiotic therapy, and predicting the prognoses in infected patients [10,11].

The association between PCT levels and residual renal function found in the current study was also consistent with the existing knowledge. PCT is demonstrated to be eliminated through the urine by kidneys [26], and the renal clearance of PCT significantly reduced in parallel to the decrease in eGFR [27,28]. As a result, elevated PCT levels could be seen in CKD patients [29].

To date, serum PCT levels have been found to elevate in AKI patients compared to those without AKI in various clinical settings [30,31]. The current study not only had findings consistent with the existing evidence but also further demonstrated the increasing PCT levels along with the increasing AKI severity irrespective of the existence of infection (Figure 3 and Table S1). The potential explanations for the association between PCT and AKI are as follows: (1) The direct cytotoxic effect of PCT on mesangial cells, which damages the kidney [32]. (2) The role of PCT as a yet unknown factor in the pathogenesis of AKI [4]. (3) The pro-inflammatory nature of AKI and the situation causing AKI might induce the synthesis of PCT. (4) The reduced clearance of PCT early in AKI before a significant elevation in SCr [4]. Besides, higher PCT was significantly associated with a higher level of an AKI marker, neutrophil gelatinase-associated lipocalin, an increased APACHE II score [33], and a reduced chance of AKI recovery [34]. Since AKI is considered an entity associated with inflammatory and disease severity [35], the above association provides a relevant pathophysiological implication between PCT and AKI.

### 4.2. Influence on Procalcitonin: Acute Kidney Injury vs. Infection

Regarding the complex influences on PCT levels of infection and AKI, diverse results exist. Although some investigation reported that the diagnostic accuracy of PCT for bacterial infection is not influenced by the existence of AKI [36], other studies disclosed that the infection-diagnostic ability of PCT was influenced in severe AKI [26] or even mild AKI [37].

In line with the previous studies [4,13,14,33,36], we disclosed that the presence of either infection or AKI was associated with an increased serum PCT level compared to those without the corresponding entity, while the infection had a higher substantial effect than AKI on PCT levels. This finding was clearly shown in the figures presenting with median PCT levels (Figure S2 and Figure 3). As a strength of the current study, we further compared PCT levels along with the worsening eGFR of four groups stratified by infection and AKI. The findings were almost consistent with the results of comparisons of median PCT levels. The only exception was that the higher PCT levels of the infection (−)/AKI (+) group compared to the infection (−)/AKI (−) group was not statistically significant (*p* = 0.059), and this insignificancy might be related to some unidentified bias that occurred in the range of eGFR 30–50 mL/min/1.73 m^2^. (Figure 2B).

Moreover, we found that the influence of infection and AKI on the PCT level could be synergistic (Figure 3 and Figure S2). The potential explanations for the synergistic effect include: (1) Both infection and AKI are associated with inflammatory/pro-inflammatory status which causes more PCT releasing into the circulation; (2) AKI associates with an impaired renal clearance of PCT from the circulation, which indirectly keeps the serum PCT levels high.

### 4.3. Influence on Procalcitonin: Acute Kidney Injury vs. Chronic Kidney Disease

In the current study, we found that AKI patients had significantly higher serum PCT levels than CKD patients at the same corresponding eGFR levels. Since the renal clearance ability of PCT decreased in both AKI and CKD, these results might reflect the higher amount of PCT production induced by the more inflammatory characteristic of AKI when compared to CKD.

### 4.4. Serum Procalcitonin as a Predictor for Acute Kidney Injury

The serum PCT level was found in the current study as an independent predictor for AKI in both the infected patients and non-infected patients. The finding was supported by the work of Heredia-Rodríguez et al. [37], which disclosed that AKI patients had significantly higher PCT levels than non-AKI patients irrespective of the presence of sepsis among cardiac surgical patients. The impact of PCT level on the AKI patients with infection when compared to that on the AKI patients without infection could be found in other investigations [12,31]. However, contradicting results exist, with some studies reporting that the serum PCT level failed to predict AKI occurrence in patients with sepsis [4] or influenza infection [13,14].

### 4.5. Limitations

There are several potential limitations in the current study. First, as a single-centered retrospective study, it was subject to bias. However, the finding in the current study that indicate that serum PCT significantly elevated since stage 1 AKI provides an essential clue for categorizing participants in the further prospective study to compare the exact influences of AKI and infection on serum PT levels. Second, the enrolled patients in the current study were mainly medical patients, and were highly selected by the criteria “existence of serum PCT measurements within 24 h after ICU admission.” The findings in the population might not be suitable to apply to other clinical settings, such as surgical or trauma patients or those who are not critically ill. Third, the current study evaluated the association between one measurement of serum PCT at initial admission and the development of AKI within seven days of admission. The serial changes of serum PCT levels during management in the hospitalization, although maybe relevant, were not taken into consideration. Fourth, it is controversial to present the residual renal function using eGFR calculated by the MDRD formula in AKI patients. We decided to do so because, as of yet, there is no other method available to compare residual renal function between patients with and without AKI.

Further multicentered, prospective researches may be warranted to investigate the predictive and prognostic values of PCT for AKI patients. Regarding the application of the PCT, “the PCT levels at different time points”, “various cut-points of PCT for different patient settings” or “using the change (in values or percentage) between different time points” may be potential strategies to determine the association among PCT, AKI, and infection.

## 5. Conclusions

The current study demonstrated that AKI had a more substantial influence on elevating serum PCT levels than CKD at the same residual renal function, and the serum PCT level of critically ill patients at ICU admission is an independent predictor for the development of AKI within the coming seven days, irrespective of infection among.

## Figures and Tables

**Table 1:** Comparisons of basic characteristics and clinical variables of the two groups.

	Total (*n* = 330)	Non-AKI Group (*n* = 203)	AKI Group (*n* = 127)	*p*-Value
**Basic demographic data**				
Age	70.5 ± 16.4	70.5 ± 16.5	70.6 ± 16.3	0.933
Gender, male	188 (57.0%)	119 (58.6%)	69 (54.3%)	0.444
Smoker	72 (21.8%)	42 (20.7%)	30 (23.6%)	0.752
Undertaking oral antibiotics	19 (5.8%)	13 (6.4%)	6 (4.7%)	0.524
Body mass index	21.9 ± 5.7	22.1 ± 5.5	21.5 ± 5.9	0.351
**Comorbidities**				
Coronal artery disease	73 (22.1%)	48 (23.6%)	25 (19.7%)	0.399
Congestive heart failure	47 (14.2%)	29 (14.3%)	18 (14.2%)	0.977
Peripheral artery occlusive disease	8 (2.4%)	5 (2.5%)	3 (2.4%)	0.954
Cerebral vascular accident	104 (31.5%)	61 (30%)	43 (33.9%)	0.469
Chronic lung disease	82 (24.8%)	51 (25.1%)	31 (24.4%)	0.884
Chronic kidney disease	98 (29.7%)	56 (27.6%)	42 (33.1%)	0.289
Diabetes mellitus	131(39.7%)	80 (39.4%)	51 (40.2%)	0.892
Cancer	44 (13.3%)	24 (11.8%)	20 (15.7%)	0.307
Liver cirrhosis	28 (8.5%)	16 (7.9%)	12 (9.4%)	0.619
Hypertension	189 (57.3%)	115 (56.7%)	74 (58.3%)	0.773
Charlson’s score, points	3.8 ± 2.6	3.7 ± 2.6	3.9 ± 2.6	0.460
**Culture-proven infection**	173 (52.4%)	103 (50.7%)	70 (55.1%)	0.438
**Infection source**				
Pneumonia	52 (15.8%)	25 (12.3%)	27 (21.3%)	0.030
Urinary tract infection	65 (19.7%)	40 (19.7%)	25 (19.7%)	0.997
Bloodstream infection	72 (21.8%)	38 (18.7%)	34 (26.8%)	0.085
Skin infection	13 (3.9%)	9 (4.4%)	4 (3.1%)	0.560
Other source	45 (13.6%)	28 (13.8%)	17 (13.4%)	0.916
**Clinical variables at ICU admission**				
Body temperature, °C	36.5 ± 1.2	36.6 ± 1.1	36.4 ± 1.2	0.135
Heart rate, beat/min	103 ± 24.4	101.9 ± 24.9	104.9 ± 23.5	0.282
Respiratory rate, breath/min	25.1 ± 9.1	24.7 ± 9.5	25.6 ± 8.6	0.363
Mean arterial pressure, mmHg	89.0 ± 25.5	89.9 ± 25.5	87.5 ± 25.6	0.415
Glasgow coma scale, points	10.3 ± 4.5	10.1 ± 4.6	10.7 ± 4.4	0.280
APACHE II, points	20.8 ± 8.2	20.1 ± 8.4	21.9 ± 7.9	0.051
SOFA score, points	6.9 ± 3.8	6.2 ± 3.8	8.1 ± 3.6	<0.001
With ventilator	99 (30.0%)	63 (31.0%)	36(28.3%)	0.604
With NIPPV	88 (26.7%)	53 (26.1%)	35(27.6%)	0.772
With vasopressor	112 (33.9%)	61 (30.0%)	51(40.2%)	0.059
Underwent CPR	29 (8.8%)	17(8.4%)	12(9.4%)	0.737
30-days mortality	81 (24.5%)	41 (20.2%)	40 (31.5%)	0.020

Note: Continuous variables with normal distribution and categorical variables were expressed as mean ± standard deviation and n (percentage), respectively. Statistical analyses were performed using independent *t*-test for the continuous variables with normal distribution, or chi-square test for categorical variables. Abbreviations: AKI = acute kidney injury, APACHE II = acute physiology and chronic health evaluation II, CPR = cardiopulmonary resuscitation, ICU = intensive care unit, NIPPV = noninvasive positive pressure ventilator, SOFA = sequential organ failure assessment.

**Table 2:** Comparisons of laboratory data of the two groups.

	Total (*n* = 330)	Non-AKI Group (*n* = 203)	AKI Group (*n* = 127)	*p*-Value
Procalcitonin, ng/mL	0.8 (0.02, 242.8)	0.5 (0.02, 242.8)	2.3 (0.05, 234.6)	<0.001
White blood cell, ×10^3^/mL	13.5 ± 8.7	12.8 ± 7.4	14.6 ± 10.4	0.057
Neutrophil/ Lymphocyte ratio	8.3 (0.2, 95.8)	7.0 (0.2, 95.8)	10.4 (0.2, 91.8)	0.068
Hemoglobin, g/dL	11.1 ± 2.9	11.2 ± 3.0	10.8 ± 2.8	0.321
Platelet, ×10^3^/mL	216.7 ± 114.0	219.8 ± 116.6	211.6 ± 110.1	0.527
Blood urea nitrogen, mmol/L	32.2 (5.3, 210.7)	23.4 (5.3, 210.7)	54.4 (11.0, 205.0)	<0.001
sCr, mmol/L	1.5 (0.3, 18.2)	1.0 (0.3, 15.9)	2.6 (0.4, 18.2)	<0.001
eGFR, ml/min/1.73 m^2^	44.2 (1.3, 557.8)	65.9 (1.3, 382.3)	23.5 (1.8, 557.8)	<0.001
AST, units/L	32.0 (3.4, 2236.0)	29.0 (9.0, 2236.0)	41.0 (3.4, 1027.0)	0.067
ALT, units/L	25.0 (1.0, 1891.0)	21.0 (1.0, 709.0)	36.0 (3.0, 1891.0)	0.002
Sodium, mmol/L	136.8 ± 9.2	136.7 ± 8.3	137.0 ± 10.6	0.806
Potassium, mEq/L	4.2 ± 1.1	4.1 ± 1.0	4.4 ± 1.2	0.031
Calcium, mEq/L	8.3 ± 1.0	8.3 ± 1.0	8.3 ± 1.1	0.967
PH	7.3 ± 0.1	7.4 ± 0.1	7.3 ± 0.1	0.512
HCO3, mEq/L	19.8 ± 7.8	21.2 ± 7.8	17.5 ± 7.4	<0.001
Glucose, mg/dl	225.1 ± 170.9	209.6 ± 132.9	249.8 ± 216.7	0.062
Albumin, mg/dl	3.1 ± 0.6	3.1 ± 0.6	3.0 ± 0.6	0.144
Bililubin (total), mg/dl	0.9 (0.0, 42.5)	0.9 (0.1, 15.0)	0.9 (0.0, 42.5)	0.210
Baseline SCr, mmol/L	1.0 (0.2, 11.2)	1.0 (0.2, 11.2)	1.0 (0.2, 10.6)	0.056
Delta SCr, mmol/L	0.9 ± 2.2	0.0 ± 0.9	2.4 ± 2.7	<0.001
Ratio of SCr	1.8 ± 1.6	1.0 ± 0.3	3.0 ± 2.1	<0.001

Note: Continuous variables with normal or abnormal distribution were expressed as mean ± standard deviation or median (range), respectively. Statistical analyses were performed using independent t-test for the continuous variables with normal distribution, or independent *t*-test after log transformation for the continuous variables with the abnormal distribution. Abbreviations: AKI = acute kidney injury, eGFR = estimated glomerular filtration rate, ALT = alanine aminotransferase, AST = aspartate aminotransferase, HCO3 = bicarbonate, SCr, serum creatinine.

**Table 3:** The independent role of procalcitonin in predicting acute kidney injury.

	Multivariate Logistic Regression		
	Odds Ratio	95% Confidence Interval	*p*-Value
Total cohort (*n* = 330)	1.27	1.12–1.43	<0.001
Non-infection group (*n* = 157)	1.38	1.12–1.71	0.003
Infection group (*n* = 173)	1.23	1.03–1.46	0.020

**Note:** All the three sets of multivariate analyses adjusted to age, gender, baseline estimated glomerular filtration rate, and sequential organ failure assessment score at intensive care unit admission. Procalcitonin was analyzed after log transformation.

## Supplementary Materials

The following are available online at https://www.mdpi.com/2077-0383/9/6/1939/s1, Figure S1. Comparisons of PCT levels between two groups stratified by infection in plots with (A) serum creatinine ratio and (B) eGFR as X-axis. Abbreviations: eGFR = estimated glomerular filtration rate; PCT = procalcitonin, Figure S2. Median levels of serum PCT among four groups stratified by AKI and infection. Note: Data were expressed as median (range). *** and ### denote *p* < 0.001 in the comparisons of serum PCT between AKI (+) and AKI (−) groups and between infection (+) and infection (−) groups, respectively. The statistical analyses were made using independent t-test. Abbreviations: AKI = acute kidney injury; PCT = procalcitonin. Table S1. Comparisons of PCT levels among patients with different AKI stages and infection states. Note: The data were presented as “PCT levels (95% confidence interval)”. The PCT levels were transformed back from the log form which had been used for analyses. The analysis of covariance was performed using mixed linear models with log-transformed PCT as the measure of interest (dependent variable), AKI states and infection as fixed factors, and baseline estimated glomerular filtration rate and sequential organ failure assessment score as covariates. 1 denotes overall comparisons among AKI stages. 2 denotes overall comparisons between infection group and non-infection group. Abbreviations: AKI = acute kidney injury, PCT = procalcitonin.
