# Supplementary material for: Independent Predictive Ability of Procalcitonin of Acute Kidney Injury among Critically Ill Patients
Source: J Clin Med. 2020 Jun 21;9(6):1939. doi: 10.3390/jcm9061939 (PMC7355446; doi:10.3390/jcm9061939)
Supplement: Supplementary file 1 [file jcm-09-01939-s001.pdf]

Supplementary information:

Table S1. Comparisons of PCT levels among patients with different AKI stages and infection states.

AKI stages	Non-AKI	AKI stage 1	AKI stage 2	AKI stage 3	[1] p-value
**Non-infection group**	0.41 (0.30-0.58)	0.72 (0.42-1.23)	1.57 (0.82-2.97)	1.24 (0.67-2.29)	<0.001
**Infection group**	1.42 (1.04-1.95)	2.46 (1.42-4.26)	5.36 (2.88-9.97)	4.25 (2.32-7.80)	
[2] **p-value**	<0.001				

Note: The data were presented as "PCT levels (95% confidence interval)." The PCT levels were transformed back from the log form, which had been used for analyses. The Analysis of Covariance was performed using mixed linear models with log-transformed PCT as the measure of interest (dependent variable), AKI stages and infection as fixed factors, and baseline estimated glomerular filtration rate and sequential organ failure assessment score as covariates.

[1] denotes overall comparisons among AKI status.

[2] denotes overall comparisons between the infection group and the non-infection group.

Abbreviations: AKI= acute kidney injury, PCT= procalcitonin.

(A)

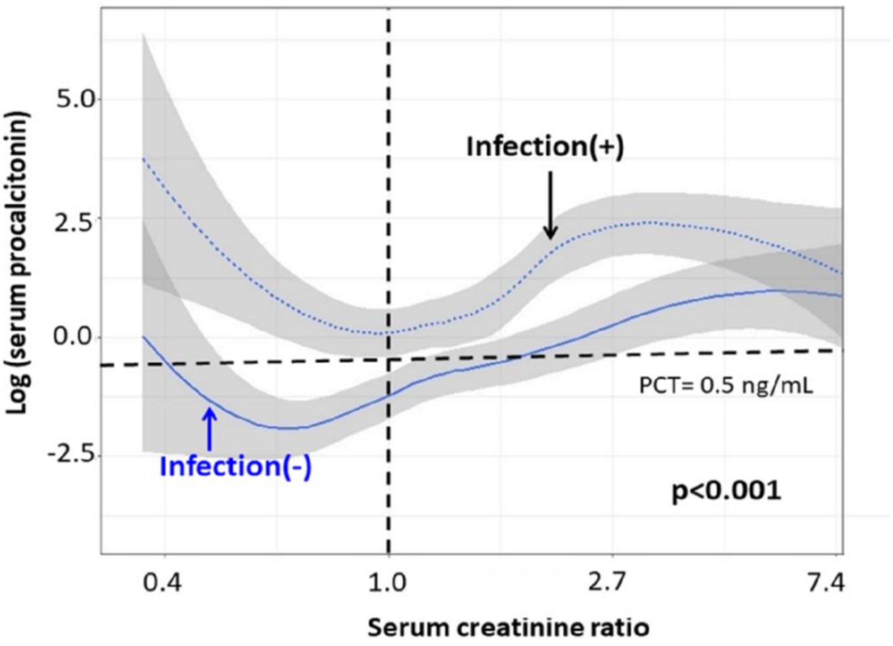

(B)

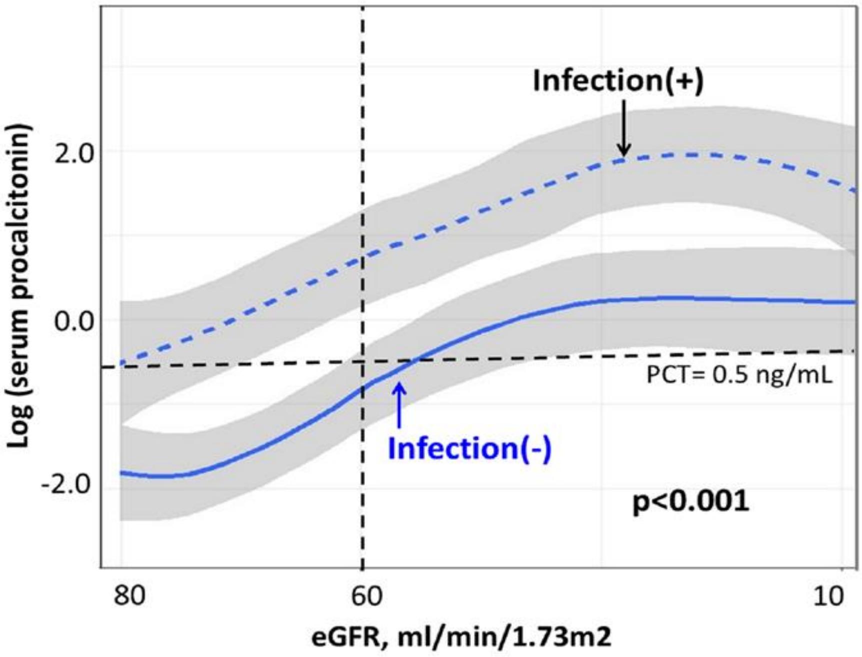

Figure S1. Comparisons of PCT levels between two groups stratified by infection in plots with (A) serum creatinine ratio and (B) eGFR as X-axis. **Abbreviations:** eGFR= estimated glomerular filtration rate; PCT= procalcitonin.

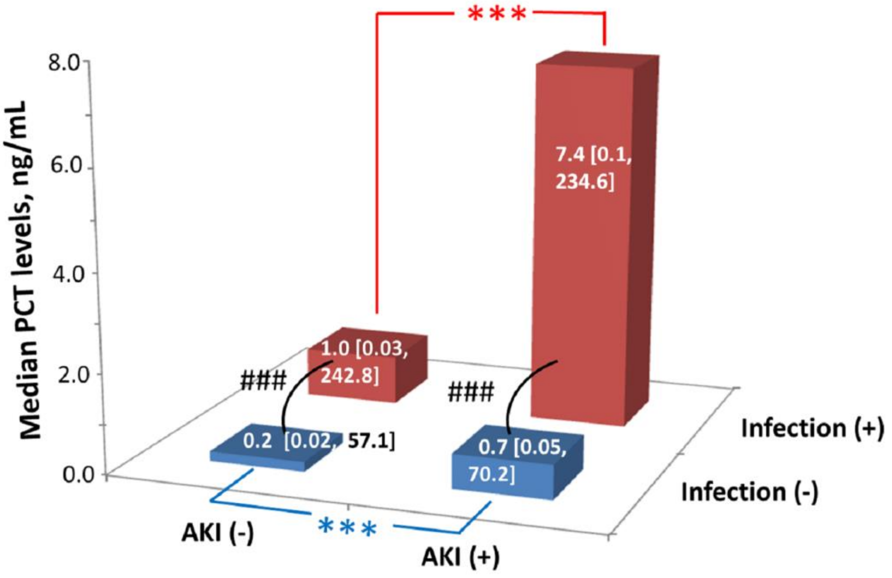

Figure S2. Median levels of serum PCT among four groups stratified by AKI and infection. Note: Data were expressed as median [range]. *** and ### denote p<0.001 in the comparisons of serum PCT between AKI(+) and AKI(-) groups and between infection(+) and infection(-) groups, respectively. The statistical analyses were made using independent t-test**; Abbreviations:** AKI= acute kidney injury; PCT= procalcitonin.
